# Predicted Immune-Related Genes and Subtypes in Systemic Lupus Erythematosus Based on Immune Infiltration Analysis

**DOI:** 10.1155/2022/8911321

**Published:** 2022-07-12

**Authors:** Lin Xu, Xiaoyan Su, Zhongcheng Liu, Aihong Zhou

**Affiliations:** ^1^Department of Nephrology, The Affiliated Taian City Centeral Hospital of Qingdao University, Tai'an 271000, Shandong Province, China; ^2^Intensive Care Unit, The Affiliated Taian City Centeral Hospital of Qingdao University, Tai'an, Shandong Province, China; ^3^Department of Neurosurgery, The First People's Hospital of Taian, Tai'an city, Shandong Province, China; ^4^Department of Rheumatology Immunology, The Second Affiliated Hospital of Shandong First Medical University, Shandong Province, China

## Abstract

**Objective:**

The present investigation is aimed at identifying key immune-related genes linked with SLE and their roles using integrative analysis of publically available gene expression datasets.

**Methods:**

Four gene expression datasets pertaining to SLE, 2 from whole blood and 2 experimental PMBC, were sourced from GEO. Shared differentially expressed genes (DEG) were determined as SLE-related genes. Immune cell infiltration analysis was performed using CIBERSORT, and case samples were subjected to *k*-means cluster analysis using high-abundance immune cells. Key immune-related SLE genes were identified using correlation analysis with high-abundance immune cells and subjected to functional enrichment analysis for enriched Gene Ontology Biological Processes and KEGG pathways. A PPI network of genes interacting with the key immune-related SLE genes was constructed. LASSO regression analysis was performed to identify the most significant key immune-related SLE genes, and correlation with clinicopathological features was examined.

**Results:**

309 SLE-related genes were identified and found functionally enriched in several pathways related to regulation of viral defenses and T cell functions. *k*-means cluster analysis identified 2 sample clusters which significantly differed in monocytes, dendritic cell resting, and neutrophil abundance. 65 immune-related SLE genes were identified, functionally enriched in immune response-related signaling, antigen receptor-mediated signaling, and T cell receptor signaling, along with Th17, Th1, and Th2 cell differentiation, IL-17, NF-kappa B, and VEGF signaling pathways. LASSO regression identified 9 key immune-related genes: DUSP7, DYSF, KCNA3, P2RY10, S100A12, SLC38A1, TLR2, TSR2, and TXN. Imputed neutrophil percentage was consistent with their expression pattern, whereas anti-Ro showed the inverse pattern as gene expression.

**Conclusions:**

Comprehensive bioinformatics analyses revealed 9 key immune-related genes and their associated functions highly pertinent to SLE pathogenesis, subtypes, and identified valuable candidates for experimental research.

## 1. Introduction

Systemic lupus erythematosus (SLE) is a chronic autoimmune disease, which predominantly affects women in childbearing age, with a wide range of clinical manifestations and incidence ranging from 1.5 to 11 per 100,000 individuals [1]. SLE remains a leading cause mortality and morbidity in young women [2, 3]. Albeit the 10-year survival rates of SLE have improved in the recent decades, mortality rates in SLE affected individuals have remained high. Several factors are recognized as associated with the variation in the incidence, course, and disease burden of SLE, including age, gender, geographic, and ethnicity variation [1–3]. Aberrant innate and adaptive autoimmune responses in SLE are driven by multiple immune cell types, inflammatory mediators, and cytokines, whereby tissue and organ injury occurs via autoantibodies and immune complex formation [4, 5]. Current treatment measures including the mainstay corticosteroids, antimalarial drugs, and immunosuppressant drugs [6] are limited in their efficacy in improving patients' quality of life, functioning, and arresting disease progression [7, 8]. As immune perturbations are the core of SLE pathology, the development of novel therapeutics and genetic and epigenetic biomarkers demands a detailed understanding of immune-related molecular regulatory processes that underlie SLE.

Large-scale analysis of genome wide association and gene expression data revealed SLE susceptibility genes display upregulation of transcription factors that leads to dysregulated gene expression networks in multiple cells involved in innate and adaptive immune responses [9]. Therefore, the recognition of deregulated gene expression patterns in immune cell types in SLE assumes crucial research significance. Accruing evidence has highlighted the role of aberrant gene expression in B and T lymphocytes and innate immune cells including platelets and neutrophils in SLE pathology [10, 11]. Emerging large-scale genetic and gene expression datasets have highlighted significant molecular heterogeneity in SLE [12]. Gene subsets associated with disease activity, progression, and subtype have been documented [13–15]. The characterization of immune subtypes of SLE can drive precision medicine approaches to improve disease diagnostics and therapy [15]. Bioinformatics immune infiltration analysis estimates immune cell abundances based on gene expression data and is a valuable tool to understand immune-cell population-related gene expression patterns in various diseases including autoimmune disease and cancer [16, 17].

Presently, immune cell infiltration patterns and associated deregulated gene expression networks in various immune cell subsets in SLE are not well understood [15]. Immune infiltration analysis can also enable identification of immune-related molecular subtypes in disease [15]. Several earlier bioinformatics studies of SLE gene expression data [18–21] have highlighted immune function associated genes among key candidates. An earlier bioinformatics study has identified the role of immune cell infiltration in SLE [22]. Therefore, the present investigation is aimed at utilizing comprehensive bioinformatics approaches to predict immune-related genes and subtypes in SLE based on immune infiltration analysis.

## 2. Material and Method

### 2.1. Datasets

Datasets for Systemic Lupus Erythematosus (SLE) were downloaded from the GEO database (http://www.ncbi.nlm.nih.gov/). Four datasets (GSE112087, GSE72509, GSE121239, and GSE50772) were obtained, of which GSE112087 and GSE72509 were from whole blood (WB) and the experimental type was obtained using high-throughput sequencing. GSE121239 and GSE50772 were from PBMC, and the experimental type belonged to array. Among them, the SLE disease samples were considered as cases, and the normal samples as control. The details of the 4 datasets are shown in Table 1.

### 2.2. Data Preprocessing

The gene expression value of GSE112087 was in Count, while the gene expression value of GSE72509 was in RPKM (Reads Per Kilobase of exon model per Million mapped reads). In order to unify the values, we converted the count of GSE112087 to RPKM with the following formula:

(1) $$\begin{array}{c} \text{RPKM}=\frac{\text{Exon mapped reads}*10^{9}}{\text{Total mapped reads}*\text{exon length}}. \end{array}$$

First, we downloaded the gene annotation file from GENCODE (http://ftp.ebi.ac.uk/pub/databases/gencode/Gencode_human/release_22/gencode.v22.annotation.gff3.gz/). We converted the expression value counts of GSE112087 gene into RPKM according to the length of the exon in the annotation file and formula (1). Then, we converted the Ensemble ID in the dataset to Gene Symbol according to the annotation file. When multiple ENSG IDs were mapped on one gene, the mean expression values of ENSG ID in samples were taken as the gene expression value. For GSE72509, we selected those genes whose TYPE was protein_coding. GSE112087 and GSE72509 were merged based on the intersection genes, and the expression values were converted to log 2 (log2) after the datasets were merged. Next, we used the ComBat method in the sva package of R project to process the log2-transformed data to eliminate the batch effect generated after data merging. For the whole blood data of SLE, we labeled the dataset obtained after removing batch effects as SLE_WB.

For PBMC data (GSE121239 and GSE5077), we converted probe ID to Gene Symbols according to the respective platform information of the datasets. When multiple probe IDs were mapped to one gene, the mean expression value of the probe ID in the samples was taken as the gene expression value. Then, we merged the two datasets of PBMC based on the common genes and used the ComBat method to eliminate the data batch effect and finally marked the merged data as SLE_PBMC.

In SLE_WB and SLE_PBMC, if the number of samples with an expression value of 0 for a particular gene exceeded 50% of the total number of samples, we removed that gene from the dataset.

### 2.3. Differentially Expressed Gene (DEG) Analysis

We performed differential expression analysis on both the SLE_WB and SLE_PBMC datasets using the “limma” package in R. For SLE_WB, we selected genes with adjusted *p* value < 0.01, ∣log2(FC) | ≥0.3 as DEG. For SLE_PBMC, genes with adjusted *p* value < 0.05, ∣log2(FC) | ≥0 were selected as DEG.

### 2.4. Predicting SLE-Related Genes

We extracted the common DEG for SLE_WB and SLE_PBMC data, and these common genes were predicted as SLE-related genes. Genes upregulated in both SLE_WB and SLE_PBMC were predicted as SLE-related upregulated genes, and genes downregulated in both SLE_WB and SLE_PBMC were predicted as SLE-related downregulated genes. We used the “pheatmap” package in R to draw heatmaps for observing differences in the expression levels of SLE-related between sample types.

### 2.5. Enrichment Analysis for the SLE-Related Gene

We used the clusterProfiler package in R project to perform GO Biological process and KEGG pathway analysis on the SLE-related genes.

### 2.6. CIBERSORT Immune Cell Infiltration Analysis

CIBERSORT (https://cibersortx.stanford.edu/) uses imputed gene expression profiles and provides an estimation of the abundances of member cell types in a mixed cell population, using gene expression data. CIBERSORT provided a gene expression signature set LM22 for 22 immune cell subtypes (Table S1). We extracted the expression values of case samples of SLE-related genes in SLE_WB and SLE_PBMC each and then combined with the immune cell gene expression feature set LM22 to perform CIBERSORT immune infiltration analysis. Cell abundance scores for the 22 immune cell subtypes in each sample were thus obtained.

We selected samples with *p* value < 0.05 and correlation coefficient > 0.75 as the significantly and strongly correlated samples of the immune cell feature set matrix and SLE expression matrix. A heatmap was constructed to check the fractional distribution of these 22 immune cells in significant samples, and Pearson's correlation analysis was applied to screen the immune cells with high abundance scores.

### 2.7. Clustering Samples Using High-Abundance Immune Cells

We performed *k*-means cluster analysis on case samples of SLE_WB and SLE_PBMC based on the high-abundance immune cells. First, we used the fviz_nbclust function of the “factoextra” package in R to determine the optimal number of clusters. The fviz_nbclust function includes three methods: average silhouette width, gap statistics, and elbow method. We combined the results of the three methods to determine the optimal number of clusters, followed by *k*-means cluster analysis on SLE_WB and SLE_PBMC. Wilcoxon's test was performed to confirm the clustering effect conducted on immune cell data, based on sample clusters.

### 2.8. Immune-Related SLE Gene Screening

For SLE_WB and SLE_PBMC, firstly we obtained the abundance values of high-abundance immune cells and the expression values of SLE-related genes in the case samples. Pearson's correlation test was applied to test the correlation between immune cells and SLE-related genes. Absolute values of all correlation statistics were used to obtain the third quartile (Q3) as the threshold and relationship pairs with ∣correlation | ≥Q3 were considered significant relationship pairs to determine SLE-related genes that were highly related to immune cells. These genes were marked as immune-related SLE genes, and the expression values in case samples were extracted. We then analyzed the differences of immune-related SLE genes in different clusters using ANOVA analysis. Genes with *p* value < 0.01 were screened as significantly different genes.

Using the above methods, we obtained differentially expressed immune-related SLE genes in the clusters of SLE_WB and SLE_PBMC cases. The intersection of these DEGs was obtained. Such genes displayed a significant relationship with immune cells in both SLE_WB and SLE_PBMC, and thus, we marked these as key immune-related SLE genes.

We used R's clusterProfiler package to analyze GO Biological process and KEGG pathways enriched in the key immune-related SLE genes, and selected functions with *p* value < 0.05 as significant functions. Protein-protein interaction (PPI) relationship pairs were obtained from the HPRD database (http://www.hprd.org/) and the BIOGRID database (http://thebiogrid.org/). PPI data obtained from the two databases were combined, and the genes that interacting with the key immune-related SLE gene were identified. Cytoscape software was used to construct the PPI network related to key immune-related SLE genes, and the network topology was analyzed.

### 2.9. LASSO Regression Analysis for Key Immune-Related SLE Genes

LASSO (Least absolute shrinkage and selection operator) Logistic Regression was applied to further screen key immune-related SLE genes. Lasso regression is a machine learning technique that identifies variables and a model that produces the least prediction error [23].

Firstly, the gene expression values of key immune-related SLE genes in case samples in SLE_WB and SLE_PBMC were extracted, and then based on the respective *k*-means cluster analysis results, LASSO was used to establish model feature screening. A refined LASSO model was obtained by constructing a penalty function, so as to facilitate the selection of key genes. The intersection of the characteristic genes obtained from SLE_WB and SLE_PBMC was obtained and was marked as significant SLE-related genes. The extracted gene expression value of these significant SLE-related genes in SLE_WB and SLE_PBMC was extracted, and boxplots were constructed to view the distribution of expression values of these genes. Wilcoxon's test was performed to analyze the differences in significant SLE-related genes in the different clusters in SLE_WB and SLE_PBMC (case vs. control, cluster1 vs. cluster2).

### 2.10. Relationship between Clinicopathological Characteristics and Significant SLE-Related Genes

The pathological characteristics of case samples in the dataset were obtained, and expression values of significant SLE-related genes in these samples correlating with pathological characteristics were obtained using Pearson's correlation analysis, in order to analyze the relationship between significant SLE-related genes and clinicopathological features.

## 3. Results

### 3.1. Differential Expression Gene

**Figure 1:**
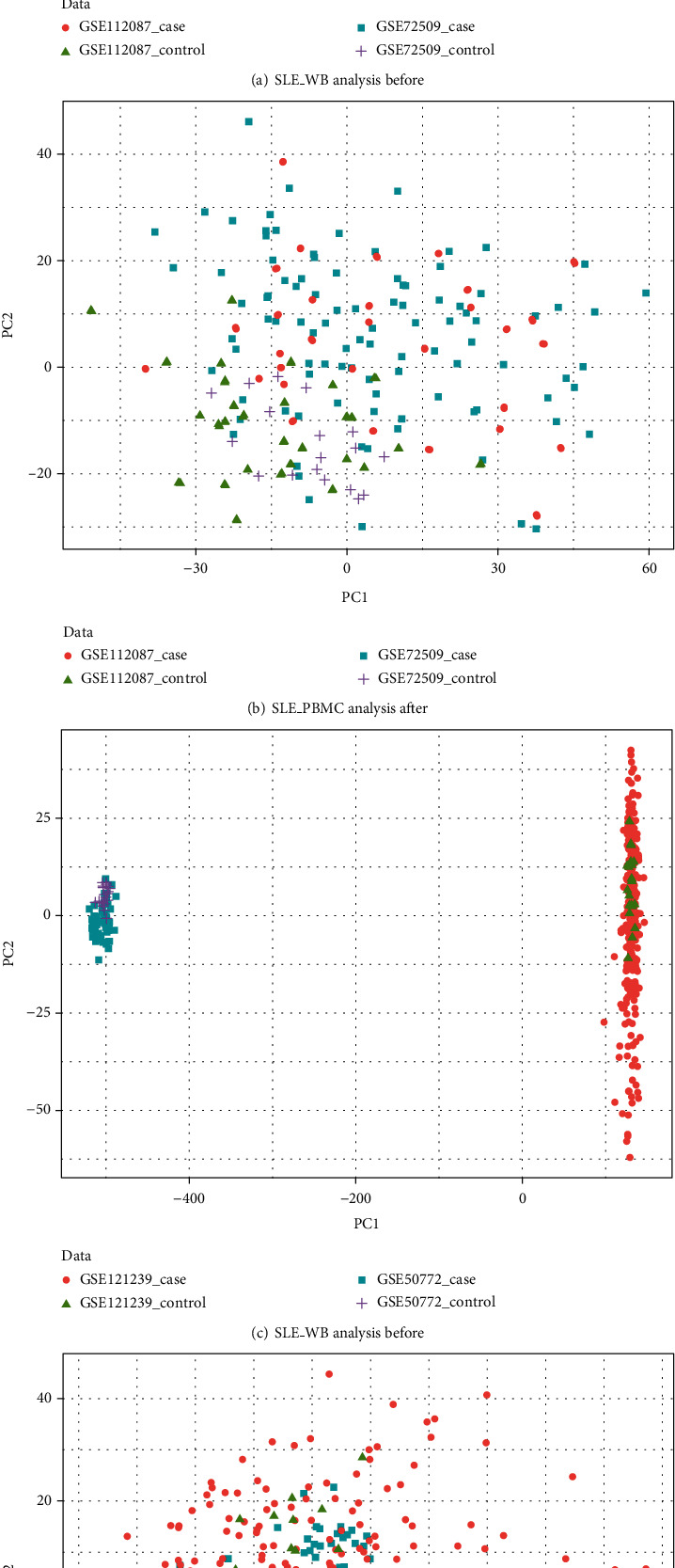
PCA analysis results of batches before and after rectification. (a, b) SLE_WB; (c, d) SLE_PBMC.

After obtaining the SLE_WB and SLE_PBMC datasets, PCA analysis was performed on the datasets before and after eliminating the batch effect, and a scatter plot depicted the analysis results (Figure 1).

As evident in Figure 1, a notable difference existed between the datasets before eliminating the batch effect, which was significantly reduced after eliminating the batch effect.

**Figure 2:**
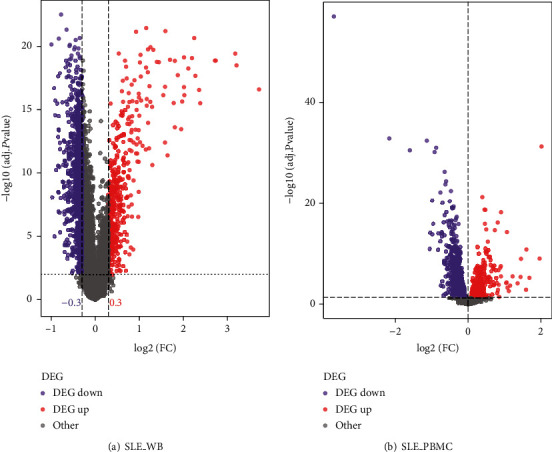
Volcano plot of differentially expressed genes in SLE_WB and SLE_PBMC. (a) Volcano plot of differentially expressed genes in SLE_WB; (b) volcano plot of differentially expressed genes in SLE_PBMC.

For SLE_WB genes with adjusted *p* value < 0.01, ∣log2(FC) | ≥0.3 indicated upregulated genes, and log2(FC) < −0.3 indicated downregulated genes. For SLE_PBMC, genes with adjusted *p* value < 0.05, and ∣log2(FC) | ≥0 indicated DEGs, where log2(FC) > 0 indicated upregulated genes, and log2(FC) < 0 indicated downregulated gene. The statistics for differential genes are shown in Table 2, and a volcano plot depicted the distribution of differential genes in the two datasets (Figure 2).

### 3.2. Predicting SLE-Related Genes

**Figure 3:**
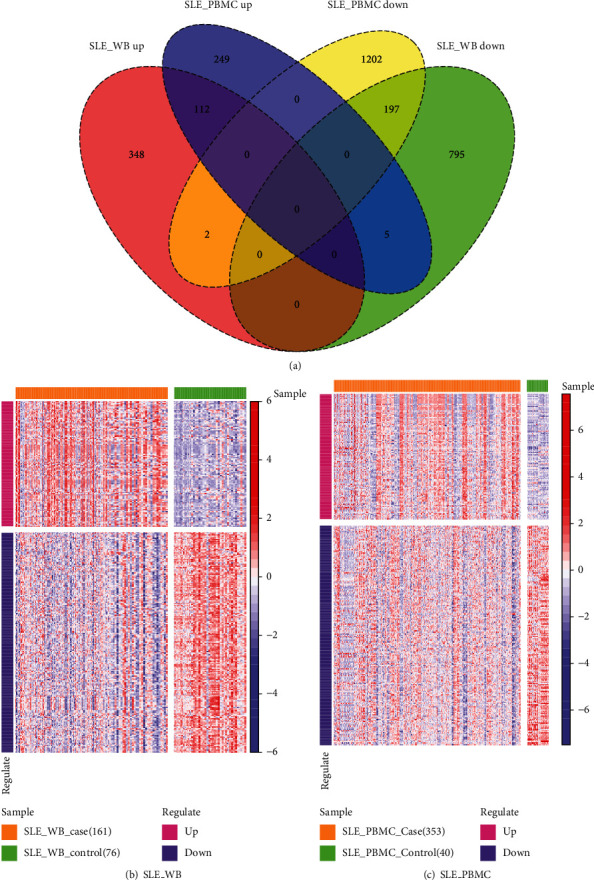
(a) Differential genes in SLE_WB and SLE_PBMC. The red and green sets in the figure indicate SLE-related genes; (b) heatmap of expression values of SLE-related genes in SLE_WB; (c) heatmap of expression values of SLE-related genes in SLE_PBMC.

1459 DEGs in SLE_WB and 1767 DEGs in SLE_PBMC were identified, with 316 intersecting genes (Figure 3(a)). Among the 316 intersecting genes, 309 genes had similar expression trends in SLE_WB and SLE_PBMC, including 112 common upregulated genes and 197 common downregulated genes. 309 genes were identified as the SLE-related genes. The expression values of SLE-related genes in SLE_WB and SLE_PBMC were displayed using a heatmap (Figures 3(b) and 3(c)). It can be seen from Figure 3 that the expression levels for the case cluster and the control cluster were significantly different.

**Figure 4:**
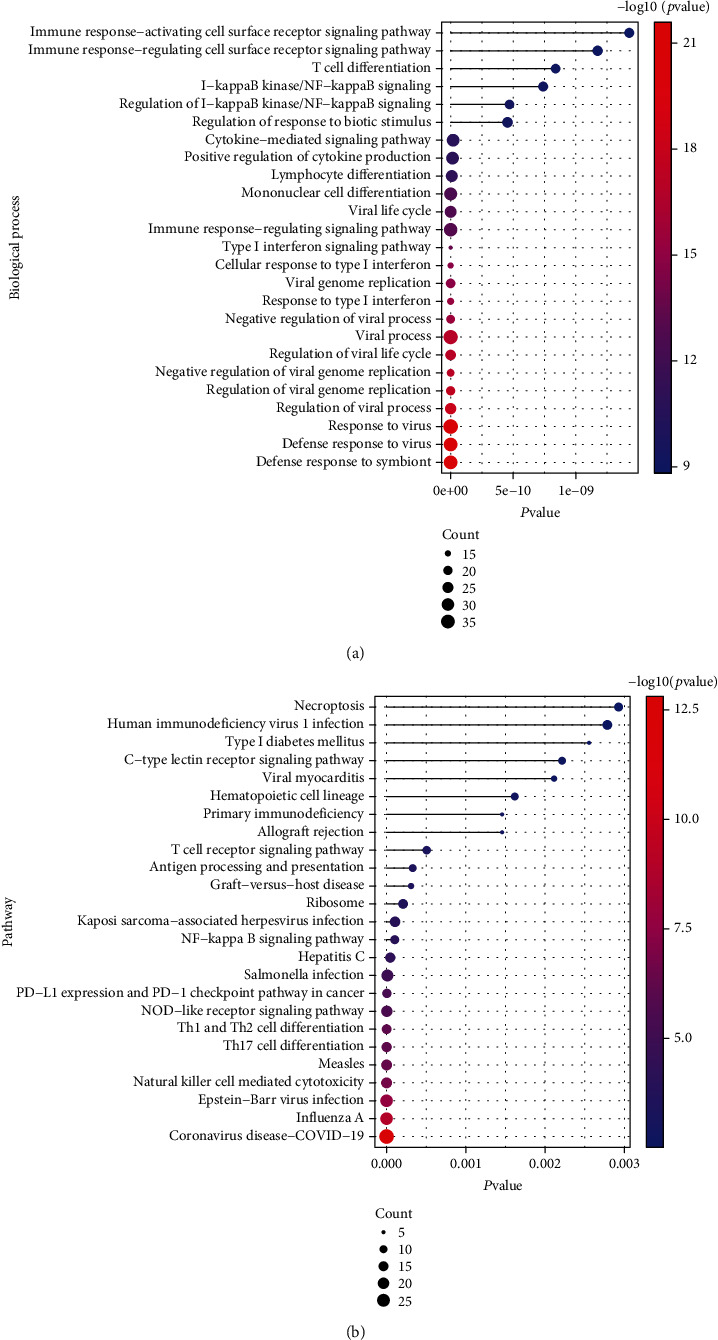
Functional enrichment for SLE-related genes (a) biological process and (b) KEGG pathway.

GO biological process and KEGG pathway analysis for these 309 SLE-related genes were performed using clusterProfiler, where *p* value <0 .05 indicated a significant pathway. The top 25 pathways are depicted in Figure 4. From the results, SLE-related genes were enriched in biological processes including defense response to virus, defense response to symbol, regulation of viral process, and negative regulation of viral genome replication (Figure 4(a)). In addition, they regulated pathways including Epstein-Barr virus infection, natural killer cell mediated cytotoxicity, NOD-like receptor signaling pathway, NF-kappa B signaling pathway, and T cell receptor signaling pathway (Figure 4(b)).

### 3.3. CIBERSORT Immune Infiltration Analysis

**Figure 5:**
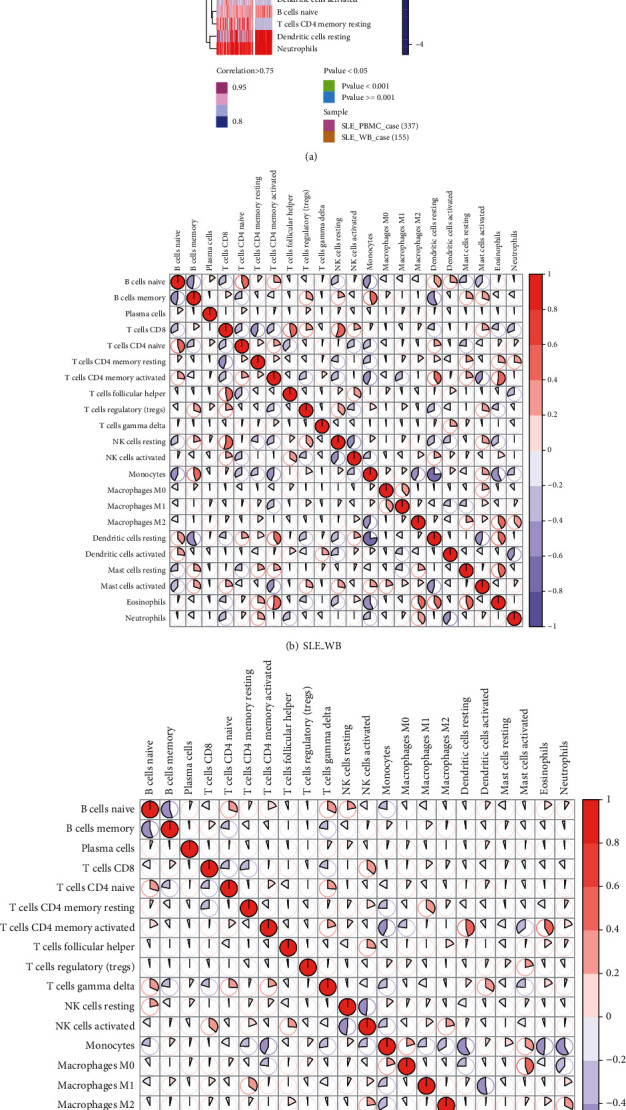
Immune cell heatmap and correlation analysis results. (a) Abundance of immune cells in SLE_WB and SLE_PBMC; (b) correlation of immune cells in SLE_WB; (c) correlation of immune cells in SLE_PBMC.

From the results of SLE_WB and SLE_PBMC immune infiltration, we selected samples with *p* value < 0.05 and correlation > 0.75 as significantly correlated samples. 155 SLE_WB disease samples and 337 SLE_PBMC disease samples were obtained, and the abundance scores of these samples in immune cells were represented (Figure 5(a)). Pearson's correlation analysis on 22 immune cells using the “corrplot” package was used to display the correlation between these immune cells as seen in Figures 5(b) and 5(c).

As seen in Figures 5, 5 immune cell types (monocytes, naive B cells, dendritic cells resting, neutrophils, and T cells CD8) were abundant in SLE_WB and SLE_PBMC and were highly correlated with other cells.

### 3.4. Clustering Samples Using High-Abundance Immune Cells

**Figure 6:**
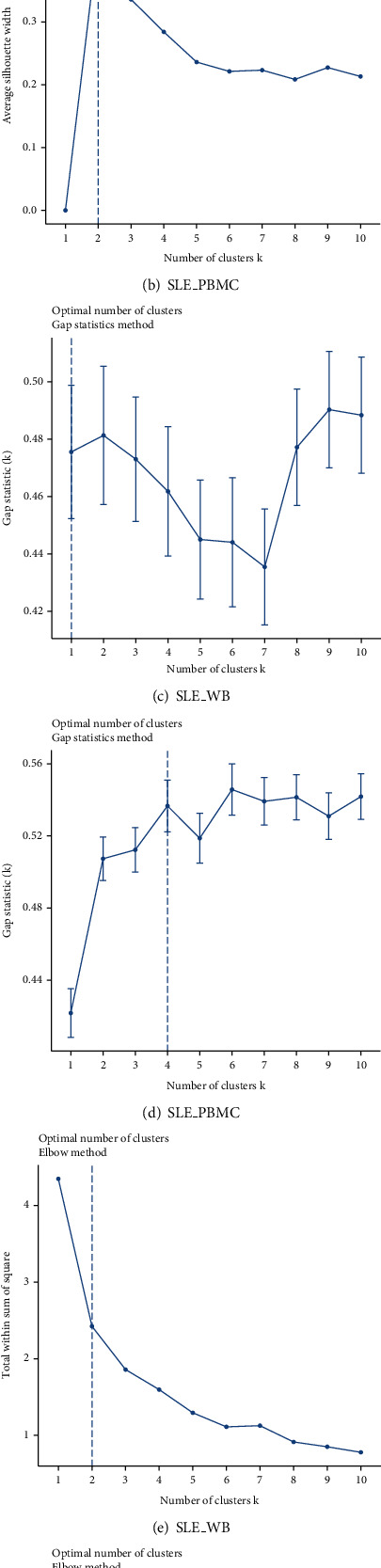
The three methods of average silhouette width, gap statistics and elbow method determine the optimal number of clusters for SLE_WB and SLE_PBMC. (a–c) Determination of the optimal number of clusters for SLE_WB by three methods.

We extracted the respective abundances of monocytes, naive B cells, dendritic cells resting, neutrophils, and T cells CD8 in SLE_WB and SLE_PBMC and then used average silhouette width, gap statistics, and elbow method to analyze the optimal number of clusters (Figures 6(a)–6(f)).

**Figure 7:**
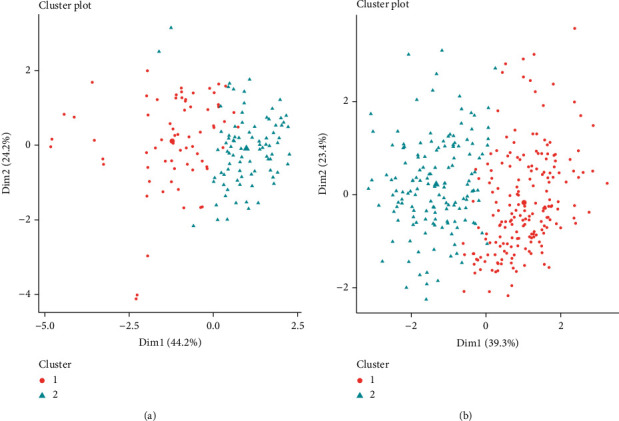
*k*-means algorithm analysis of (a) SLE_WB and (b) SLE_PBMC results.

The optimal number of clusters for the three methods of average silhouette width, gap statistics and elbow method in SLE_WB, showed 2 clusters, 1 cluster and 2 clusters, respectively. Using comprehensive comparison, for SLE_WB, we chose the number of clusters as 2 clusters. In SLE_PBMC, the optimal number of clusters for the three methods of average silhouette width, gap statistics and elbow method were 2 clusters, 4 clusters, and 2 clusters, respectively. Using comprehensive comparison, for SLE_PBMC, we chose the number of clusters to be 2 clusters. After determining the number of clusters, we used *k*-means to analyze the data. A cluster plot was drawn to display the analysis results (Figures 7(a) and 7(b)).

**Figure 8:**
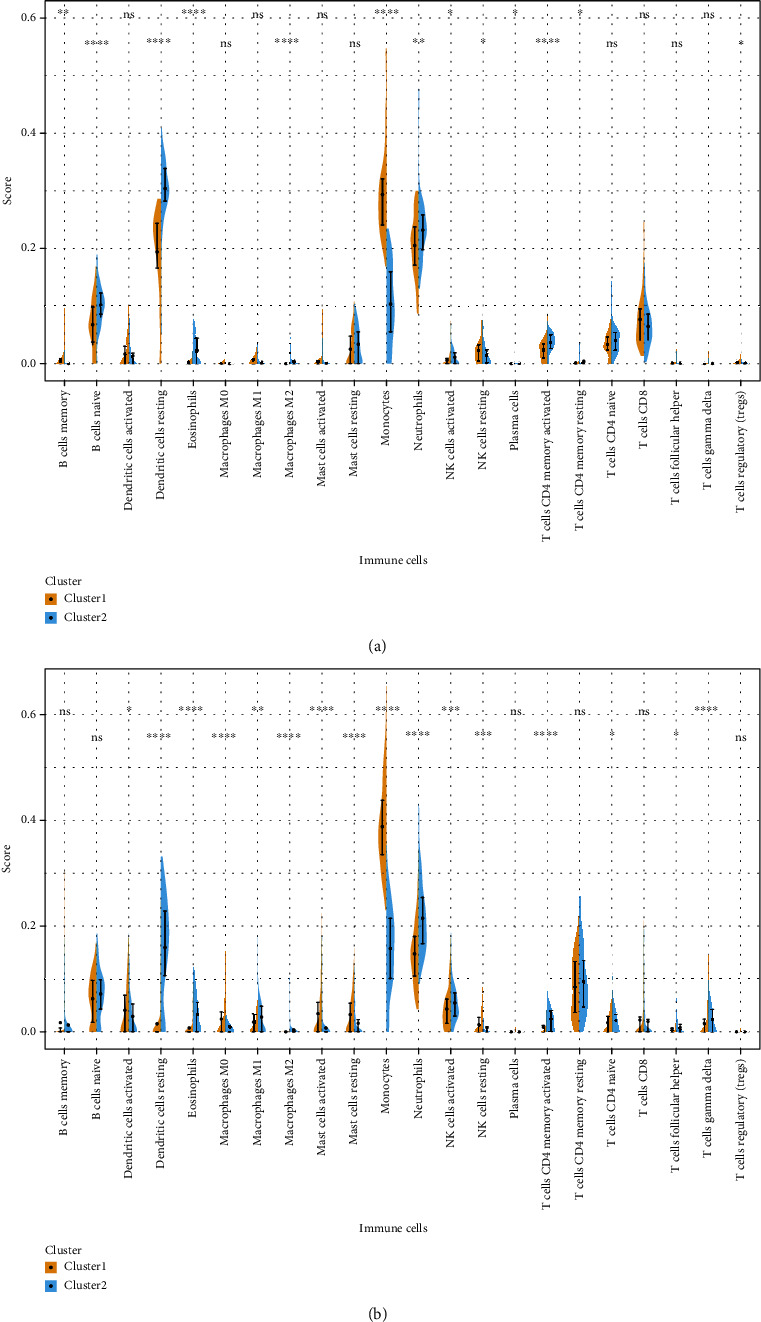
Differences of 22 immune cells in two clusters of (a) SLE_WB and (b) SLE_PBMC. ^ns^*p* > 0.05, ^∗^*p* ≤ 0.05,  ^∗∗^*p* ≤ 0.01,  ^∗∗∗^*p* ≤ 0.001, and^∗∗∗∗^*p* ≤ 0.0001.

Wilcoxon's test to examine the differences of 22 types of immune cells in different clusters is depicted in Figures 8(a) and 8(b).

From the results, monocytes, dendritic cells resting, and neutrophils were significantly different in the two clusters of SLE_WB and SLE_PBMC.

### 3.5. Immune-Related SLE Gene Screening

**Figure 9:**
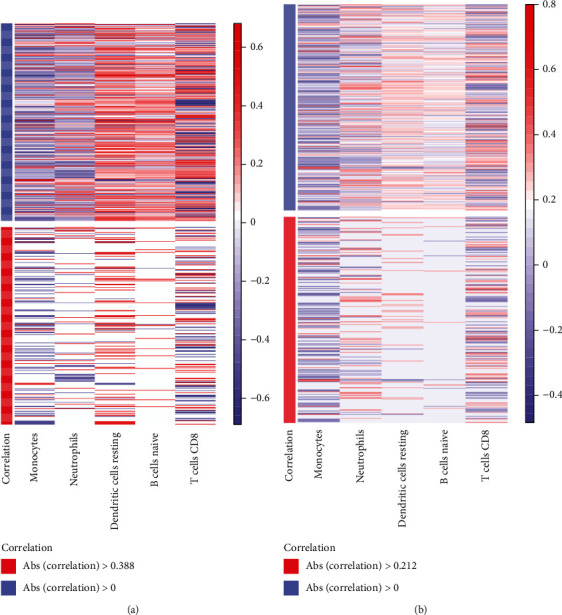
Correlation analysis of high abundance immune cells and SLE-related genes in (a) SLE_WB and (b) SLE_PBMC.

The outcomes of Pearson's correlation analysis of 5 highly abundant immune cells and 309 SLE-related genes are depicted in Figures 9(a) and 9(b). In addition, we extracted genes with higher correlation with immune cells based on correlation value.

∣Correlation(Q3) | = 0.388 for immune cells and SLE-related genes in SLE_WB and ∣Correlation(Q3) | = 0.212 in SLE_PBMC are evident in Figure 9. We extracted relation pairs with correlation values greater than |Correlation(Q3)| in SLE_WB and SLE_PBMC, as significant relation pairs. Using the genes in the significantly related pairs of SLE_WB and SLE_PBMC and then taking the intersection of the genes, we finally obtained a total of 204 SLE-related genes. We then performed ANOVA analysis based on expression values of 204 genes in SLE_WB and SLE_PBMC with 2 clusters. Genes with *p* < 0.01 were considered as the significant DEGs, and the intersection of DEGs was obtained from SLE_WB and SLE_PBMC. These intersecting genes were marked as immune-related SLE genes, and 65 immune-related SLE genes were thus acquired.

**Figure 10:**
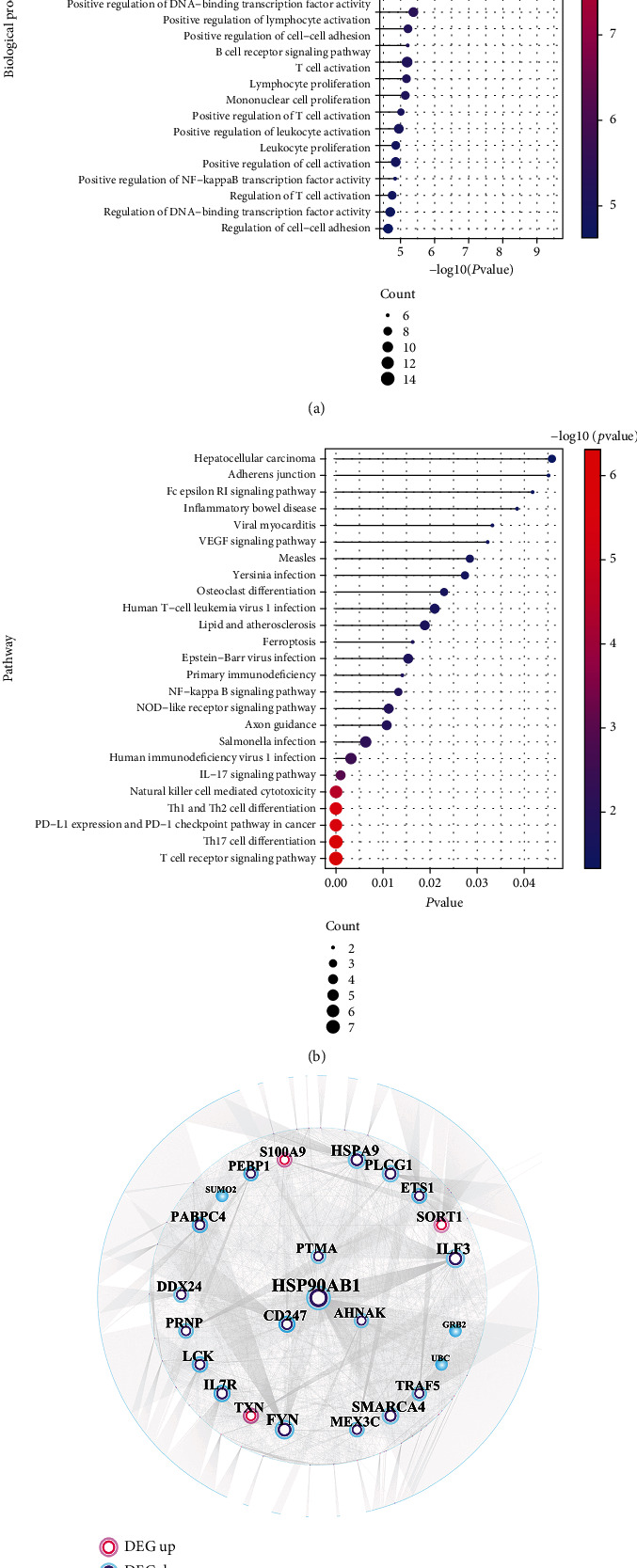
Functional enrichment analysis results of 65 immune-related SLE genes. (a) Biological process significantly enriched by immune-related SLE genes; (b) KEGG pathway significantly enriched by immune-related SLE genes; (c) the PPI network of immune-related SLE genes.

GO biological process and KEGG pathway analysis on these 65 immune-related SLE genes results are depicted. We selected features with a *p* value < 0.05 and displayed the top 25 pathways in Figures 10(a) and 10(b). Immune-related SLE genes were mainly involved in biological processes including immune response-activating cell surface receptor signaling pathway, immune response-regulating signaling pathway, antigen receptor-mediated signaling pathway, and T cell receptor signaling pathway; they also regulated Th17 cell differentiation, Th1 and Th2 cell differentiation, IL-17 signaling pathway, NF-kappa B signaling pathway, and VEGF signaling pathway.

In addition, we extracted the interacting proteins of 65 immune-related SLE genes from the public databases HPRD and BIOGRID and obtained a total of 4976 PPI-related pairs. A PPI network for immune-related SLE-related genes was constructed after deduplicating the relationship pair and contained 3188 nodes and 4939 edges. Cytoscape was used analyze the topological properties of the network and extract nodes with higher degrees listed in Table 3.

For the PPI network, we hid nodes with lower degrees and selected nodes with higher degrees to display (Figure 10(c)).

### 3.6. LASSO Regression Analysis for Key Immune Related SLE Gene

**Figure 11:**
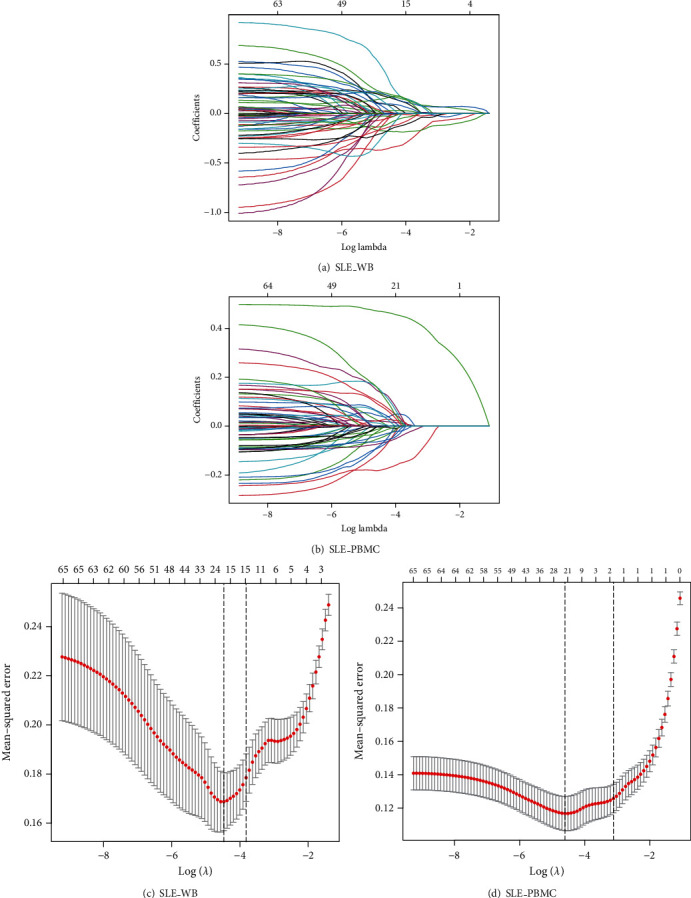
LASSO feature screening of 65 SLE-related genes. (a, b) LASSO analysis results of SLE_WB and SLE_PBMC. Each line in the figure represents a gene. When the gene tends to 0, the larger the value of the abscissa (log lambda), the more critical the gene is. (c, d) Cross-validation results of SLE_WB and SLE_PBMC models. There are two dotted lines in the figure, one is the *λ* value lambda.min when the mean square error is the smallest, and the other is the *λ* value lambda.1se which is one standard error when the mean square error is the smallest. These two values can be selected according to the analysis requirements. One, the number corresponding to the dotted line is the result of the number of screened genes.

The expression values of 65 immune-related SLE genes in case samples of SLE_WB and SLE_PBMC were extracted, and LASSO model was built (Figures 11(a)–11(d)). Nin common feature genes (DUSP7, DYSF, KCNA3, P2RY10, S100A12, SLC38A1, TLR2, TSR2, and TXN) between SLE_WB and SLE_PBMC acting as key immune-related SLE genes were identified.

### 3.7. Relationship between Clinicopathological Characteristics and Significant SLE-Related Genes

**Figure 12:**
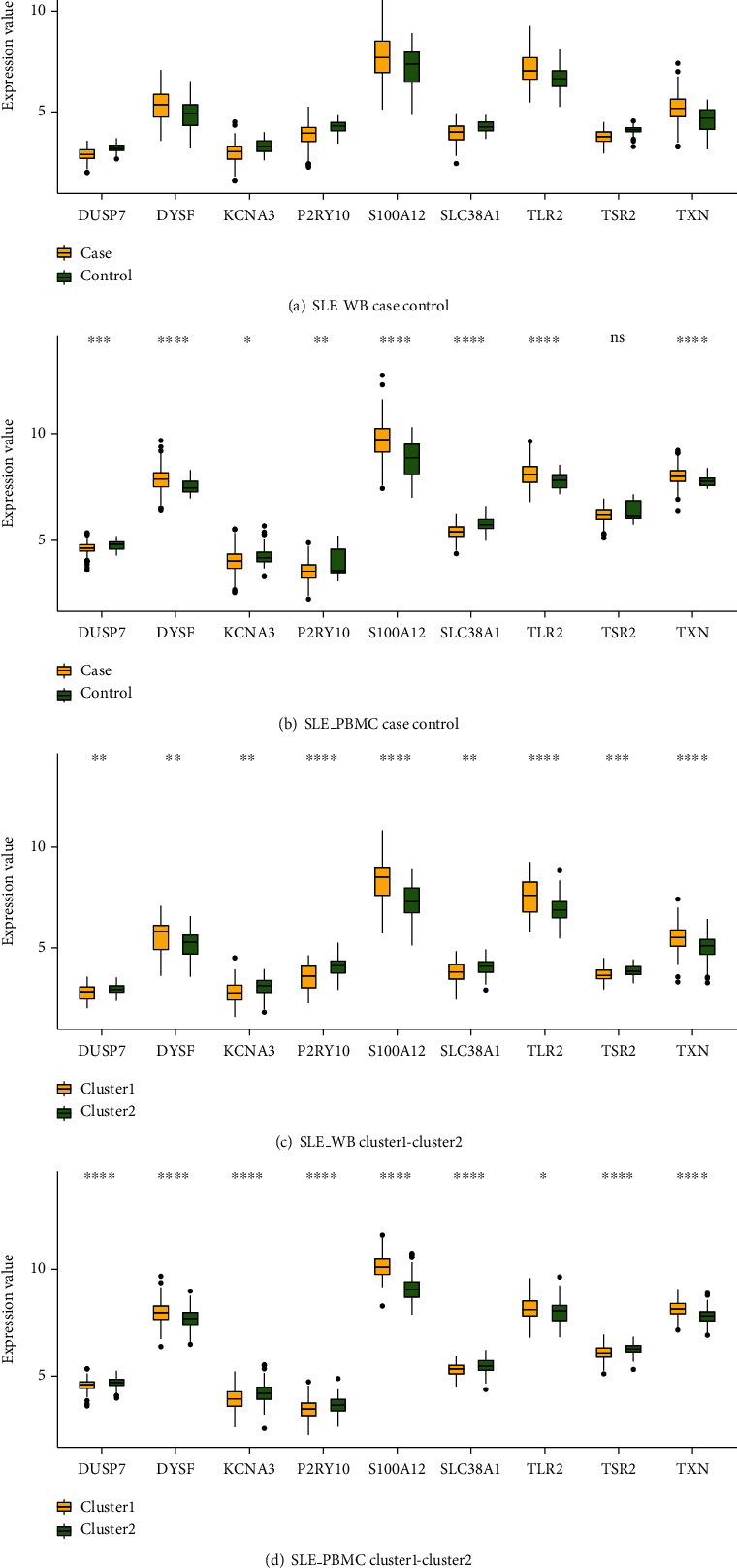
Expression levels of 9 key immune-related SLE genes in SLE_WB and SLE-PBMC. (a, b) are the expression distributions in the case group and control group for SLE_WB and SLE_PBMC, respectively. (c, d) are the expression distribution of cluster1 group and cluster2 group of SLE_WB and SLE_PBMC, respectively. ^ns^*p* > 0.05, ^∗^*p* ≤ 0.05,  ^∗∗^*p* ≤ 0.01,  ^∗∗∗^*p* ≤ 0.001, and^∗∗∗∗^*p* ≤ 0.0001.

Boxplots depicting the distribution of expression values of these 9 key immune-related SLE genes are shown (Figures 12(a)–12(d)). Wilcoxon's test using these gene expression values in the sample groups was performed showing 9 genes were significantly different in different groups.

**Figure 13:**
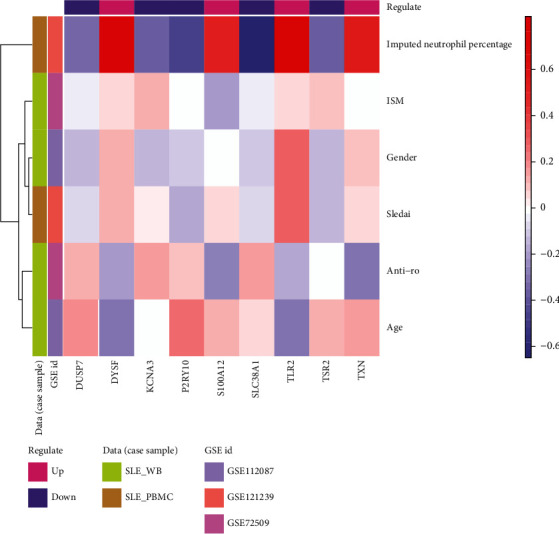
Correlations between 9 key immune-related and clinicopathological features.

In Table 1, we depict 6 clinicopathological features (age, gender, anti-Ro, ISM, sledai, and imputed neutrophil percentage) in different datasets and organized these pathological feature files and extracted case samples. For each pathological feature, we extracted the case samples' 9 key immune-related SLE gene expression levels and clinicopathological features and analyzed the relationships between them using Pearson's correlation (Figure 13).

The results showed that the imputed neutrophil percentage was highly correlated with the 9 SLE-related genes and consistent with the gene expression pattern. Relationships between other pathological features and genes were evident where anti-Ro is showed the inverse pattern with gene regulation.

## 4. Discussion

The present study used integrative and comprehensive bioinformatics approaches to identify the most significant immune-related genes in SLE and further identified case subsets based on immune-cell infiltration analysis. The main findings revealed a 9-candidate gene signature (DUSP7, DYSF, KCNA3, P2RY10, S100A12, SLC38A1, TLR2, TSR2, and TXN), which discriminated both cases from controls and identified immune cell based clusters. These genes' involvement in immune-related functions was supported by a previous literature.

DUSP (dual specificity phosphatase) genes have been found to be involved in autoimmune disease and in T cell activation [24, 25] in SLE. Dysferlin encoded by DYSF has been typically implicated in myopathies and found to regulate cell adhesion in monocytes [26]. The gene KCNA3 is associated with voltage gated Kv 1.3 potassium ion channels and has been implicated in multiple sclerosis and autoimmune pancreatitis [27, 28] Functionally, KCNA3 has been shown to regulate CD4 T memory cell function [29, 30] via Kv 1.3 channels, which are considered an emerging target in autoimmune disease [31]. P2RY10 encodes a G-protein coupled receptor activated by lysophosphatidylserine that is shown to induce chemokine induced CD4 T cell migration by RhoA activation in response to autocrine and paracrine signals [32]. P2RY10 has also been implicated in eosinophil degranulation [33] and considered a target for eosinophil associated diseases. The S100A12 gene belongs to the family of calcium binding proteins expressed in granulocytes that act as a chemoattractant and is considered a pivotal player in inflammation [34] by contributing to neutrophil and monocyte migration [35] and monocyte activation [36]. SLC38A1 belongs to the solute carrier family and is an important transporter of amino acids, which are known to be perturbed in T cells in SLE [37]. SLC38A1 transports alanine in CD4 T cells upon T cell receptor activation [38]. The role of toll like receptor 2 TLR2 has been well investigated in SLE initiation in context of pathogen recognition and autoantibody production to self-DNA antigens [39]. Furthermore, PMBC bound TLR2 has been shown as marker of disease activity in SLE [40] and SLE susceptibility has been found to be associated with TLR2 gene polymorphism [41]. TSR2 is known to inhibit nuclear factor-*κ*B transcription and induce apoptosis [42]. The TXN gene encodes thioredoxin which reacts with procaspase-3 in T cells to inhibit apoptosis [43].

Among these, the upregulated genes included DYSF, S100A12, TXN, and TLR2, which were strongly correlated with imputed neutrophil abundance, possibly indicating disease subtype that should be investigated in experimental research. In SLE, aberrant neutrophil function, including the retention of low-density neutrophils (LDN) in microvasculature, is recognized as an important mechanism of organ damage and vasculopathy, and differences in neutrophil gene expression are seen in association with neutrophil trafficking [44]. Furthermore LDN also secrete type I interferon (IFN), activating adaptive immune responses in SLE [45]. Therefore, gene signatures of neutrophil heterogeneity may be very relevant to disease subtyping in SLE. These 4 genes also showed an inverse correlation with anti Ro antibodies, which are shown to negatively correlate with complement C3 in SLE [46], which is essential for neutrophil infiltration and neutrophil extracellular trap (NET) formation [47]. In the cluster analysis using imputed high abundance immune cells, monocytes, dendritic cells resting, and neutrophils were found significantly different in representation in both PMBC and whole blood sample gene expression data, whereas Treg cells, CD 4 activated T cells, NK cells, and B cells were also different in the whole blood samples. In an earlier work using gene expression data, 3 main subtypes of SLE were determined as an interferon (IFN) subtype enriched in viral infection and IFN deregulation, a mixed subtype of more severe nature characterized by viral, bacterial and fungal infection-related modules, and a neutrophil-elastase subtype (NE) enriched in bacterial and fungal infection related modules [48]. Others have identified T cell transcriptome-based molecular subtypes of SLE including severe subtypes marked by differences in genes related to membrane protein production [49].

Functional enrichment analysis of SLE-related genes indicated several biological processes and pathways associated with defense response to viruses, which bear pathogen associated molecular patterns (PAMPS) that trigger interferon production from T cells and have a central role in SLE induction [50]. Among KEGG pathways, necroptosis, a form of caspase independent programmed cell death was the top enriched pathway. Increased interferon signaling potentiates necroptosis in SLE and may be an important means of sustained tissue damage [51]. The top GO biological process enriched in the immune-related SLE genes concerned RNA splicing, which is a fundamental process crucial to maintain diversity in protein isoforms and also regulates key immune processes such as lymphocyte differentiation and activation [52]. Analysis of RNA splicing variants can offer a venue to understand variations in gene expression networks, cell type, and localization [53]. The top among the PPI network analysis included the downregulated HSP90AB1 gene, which encodes a heat shock protein, and its polymorphism has been recently associated with SLE risk and glucocorticoid response [54] Elevated HSP90AB in the serum from SLE patients has been documented, and it is purported to be a key regulator of innate and adaptive responses via controlling signal transduction, protein folding, and transport [55].

These findings must be viewed keeping in mind the limitation of the study. Here, we analyzed a gene signature based on SLE-related DEGs that were common to whole blood and PBMC samples, which may limit the generalizability of the findings to other cell types or blood. These two datasets are not completely comparable owing to different cell types in whole blood, which may be more representative of the global immune response, and therefore, these results should be confirmed in future studies of multiple datasets from clinical samples. Of note, the application of single transcriptomics can enable granular analysis of immune cell perturbation in SLE. Furthermore, here, we have analyzed microarray data, and RNA-seq datasets should be addressed in future studies. However, the incorporation of high-throughput sequencing data and multiple sample types augments the relevance of these findings particularly to PBMC cells. The 9-immune-related gene signature of SLE and its relevance to SLE subtypes needs to be validated in experimental models and clinical research. Furthermore, clinical and pathological features of samples clustering based on high abundance immune cell types should be examined in further research. Moreover, the incorporation of data pertaining to longitudinal disease progression and drug response scores should be addressed in future research in SLE and its molecular subtypes. Clinical translation of the present data includes precision medicine approaches wherein prognosis and treatments can be better tailored based on patients' molecular subtyping and also pave the way for novel drug discovery.

## 5. Conclusion

In sum, the present study applied a series of bioinformatics analysis and identified a 9 immune-related gene signature in SLE based on immune infiltration analysis and depicted their associated clinicopathological characteristic, functionally enriched pathways, and interacting genes. These findings offer important insights into the immunological heterogeneity in SLE and its relevant cellular and molecular aspects.

## Figures and Tables

**Table 1:** Sample information in SLE dataset.

	GSE112087	GSE72509	GSE121239	GSE50772
Series type	High-throughput sequencing (count)	High-throughput sequencing (RPKM)	Array	Array
Case	62	99	292	61
Control	58	18	20	20
Total	120	117	312	81
Platform	GPL16791	GPL16791	GPL13158	GPL570
Tissue	Whole blood	Whole blood	PBMC	PBMC
Key clinical features from the dataset	(1) Age (2) Gender	(1) Anti-Ro (2) Ism	(1) Imputed neutrophil percentage	Unknown

**Table 2:** Statistics of DEG for SLE.

	SLE_WB	SLE_PBMC
Datasets	GSE112087, GSE72509	GSE121239, GSE50772
Adjusted *p* value	*p* < 0.01	*p* < 0.05
|log2(FC)|	∣log2(FC) | >0.3	∣log2(FC) | >0
DEG up	642	366
DEG down	997	1401
Total DEG	1459	1767

**Table 3:** Network topology properties of 20 nodes with high degree.

Name	regulate_array	regulate_NGS	Degree	Average shortest path length	Betweenness centrality	Closeness centrality	Clustering coefficient	Topological coefficient
HSP90AB1	Down	Down	738	2.278473	0.353518	0.43889	5.92E-04	0.003079
FYN	Down	Down	376	2.383381	0.164518	0.419572	0.002965	0.004162
ILF3	Down	Down	357	2.708694	0.139646	0.369182	5.35E-04	0.009469
HSPA9	Down	Down	282	2.771575	0.089005	0.360806	7.07E-04	0.01536
SMARCA4	Down	Down	242	2.823548	0.100178	0.354164	4.12E-04	0.017488
PLCG1	Down	Down	227	2.743022	0.074354	0.364561	0.005731	0.009125
IL7R	Down	Down	199	2.567212	0.080946	0.389528	0.005025	0.00771
CD247	Down	Down	196	2.516522	0.091615	0.397374	0.003611	0.006817
LCK	Down	Down	159	2.550529	0.045982	0.392075	0.010509	0.00904
PABPC4	Down	Down	149	2.839269	0.039722	0.352203	0.001814	0.017293
SORT1	Up	Up	134	2.923965	0.068109	0.342001	0	0.018145
TXN	Up	Up	132	2.853064	0.040495	0.3505	0.001619	0.017513
DDX24	Down	Down	124	2.686558	0.047209	0.372224	0.004196	0.010678
ETS1	Down	Down	115	2.923965	0.045398	0.342001	0	0.034622
S100A9	Up	Up	100	2.939365	0.028612	0.34021	0	0.0476
PRNP	Down	Down	95	3.091434	0.041419	0.323474	0	0.026608
PTMA	Down	Down	94	2.875842	0.030904	0.347724	0.002288	0.018951
MEX3C	Down	Down	92	2.936157	0.027107	0.340581	0	0.039171
AHNAK	Down	Down	77	2.858839	0.018477	0.349792	0.007861	0.022708
PEBP1	Down	Down	67	2.896696	0.017795	0.345221	0.002714	0.023306

## Data Availability

The datasets used and/or analyzed during the current study are available from the corresponding author on reasonable request.
